# Lung Biopsy With a Non-intubated VATS Approach in an Obese Population: Indications and Results

**DOI:** 10.3389/fsurg.2022.829976

**Published:** 2022-03-04

**Authors:** Roberto Cherchi, Paolo Albino Ferrari, Francesco Guerrera, Giulia Grimaldi, Matteo Pinna-Susnik, Alessandro Murenu, Giulio Luca Rosboch, Paraskevas Lybéris, Federica Ibba, Ludovica Balsamo, Laura Saderi, Alessandro Giuseppe Fois, Enrico Ruffini, Giovanni Sotgiu

**Affiliations:** ^1^Division of Thoracic Surgery, “A. Businco” Oncology Hospital, Azienda di Rilievo Nazionale di Alta Specializzazione (A.R.N.A.S.) “G. Brotzu”, Cagliari, Italy; ^2^Department of Surgical Science, University of Torino, Turin, Italy; ^3^Department of Thoracic Surgery, Azienda Ospedaliero Universitaria Città della Salute e della Scienza di Torino, Turin, Italy; ^4^Department of Anesthesia, Intensive Care and Emergency, Azienda Ospedaliero Universitaria Città della Salute e della Scienza di Torino, Turin, Italy; ^5^Unit of Respiratory Diseases, University Hospital Sassari Azienda Ospedaliera Universitaria (A.O.U.), Sassari, Italy; ^6^Clinical Epidemiology and Medical Statistic Unit, Department of Medical, Surgical and Experimental Science, University of Sassari, Sassari, Italy; ^7^Department of Medical, Surgical and Experimental Science, University of Sassari, Sassari, Italy

**Keywords:** non-intubated thoracoscopy, interstitial lung disease (ILD), obesity, perioperative risk factors, surgical lung biopsy (SLB)

## Abstract

**Background:**

According to the international guidelines, patients affected by interstitial lung disease with unusual clinical presentation and radiological findings that are not classic for usual interstitial pneumonia end up meeting criteria for surgical lung biopsy, preferably performed with video-assisted thoracic surgery. The growing appeal of non-intubated thoracic surgery has shown the benefits in several different procedures, but the strict selection criteria of candidates are often considered a limitation to this approach. Although several authors define obesity as a contraindication for non-intubated thoracoscopic surgery, the assessment of obesity as a dominant risk factor represents a topic of debate when minor tubeless procedures such as lung biopsy are considered. Our study aims to investigate the impact of obesity on morbidity and mortality in non-intubated lung biopsy patients with interstitial lung disease, analyzing the efficacy and safeness of this procedure.

**Materials and Methods:**

The study group of 40 obese patients consecutively collected from 202 patients who underwent non-intubated lung biopsy was compared with overweight and normal-weight patients, according to their body mass index. Post-operative complications were identified as the primary endpoint. The other outcomes explored were the early 30-day mortality rate and intraoperative complications, length of surgery, post-operative hospitalization, patient's pain feedback, and diagnostic yield.

**Results:**

The overall median age of the patients was 67.4 years (60, 73.5). No 30-day mortality or significant differences in terms of post-operative complications (*P* = 0.93) were noted between the groups. The length of the surgery was moderately longer in the group of obese patients (*P* = 0.02). The post-operative pain rating scale was comparable among the three groups (*P* = 0.45), as well as the post-operative length of stay (*P* = 0.96). The diagnosis was achieved in 99% of patients without significant difference between groups (*P* = 0.38).

**Conclusion:**

Our analysis showed the safety and efficacy of surgical lung biopsy with a non-intubated approach in patients affected by lung interstitiopathy. In the context of perioperative risk stratification, obesity would not seem to affect the morbidity compared to normal-weight and overweight patients undergoing this kind of diagnostic surgical procedure.

## Introduction

With the advent of video-assisted thoracic surgery (VATS), the outcomes of surgical lung biopsy in the diagnosis of interstitial lung disease (ILD) have remarkably improved. Nevertheless, the post-operative high mortality rates reported (1, 2) contribute to the lack of surgical lung biopsies being performed with a considerable decline in an accurate ILD diagnosis (3). Hutchinson et al. reported in-hospital mortality of 1.7% (4), but overall 30-day mortality can range between 4.3 and 17.5% following acute exacerbation of ILD after biopsy potentially triggered by the mechanical ventilation (5). In recent years, non-intubated VATS (NIVATS) procedures have been carried out to minimize the risks of general anesthesia through the avoidance of muscle relaxants and lung injuries from positive pressure ventilation, preserving an acceptable diagnostic and curative outcome. One of the most relevant aspects of this less invasive technique is the need for proper patient selection according to strict exclusion criteria for each thoracic surgical procedure. Obesity, usually regarded as a body mass index (BMI) ≥ 30, is considered a relative and expert-opinion-based contraindication in non-intubated thoracic surgery, representing a significant risk factor for conversion to intubation (6). Due to the uncontrolled cough and wide diaphragmatic excursions, especially in obese patients, even some minor non-intubated surgical procedures could become extremely demanding. The present study aims to evaluate whether obesity hinders NIVATS lung biopsy, considering risk factor-related perioperative stratification.

## Materials and Methods

### Patient Population

We conducted a retrospective review on medical records of 202 undetermined ILD patients who underwent non-intubated lung biopsies between April 2015 and November 2021 at the Division of Thoracic Surgery—Oncology Hospital “A. Businco (Cagliari-Italy) and at the Department of Thoracic Surgery”—“A.O.U. Città della Salute e della Scienza di Torino” (Turin-Italy). Informed written consent was obtained from all the patients. The study was approved by the Institutional Review Board (Reference Ethics Committee No. PG/2017/16770) and conducted in accordance with the Declaration of Helsinki (as revised in 2013). Patients were excluded from the study if any of the following were present: age <18 years, haemodynamic instability, patient already intubated and ventilated, or anticipated need for extensive decortication.

### Endpoints and Definition

Based on the aim of the study, the primary endpoint was to assess the safety of this procedure in obese patients in terms of in-hospital post-operative complications. In addition, secondary outcomes were early mortality after surgery, intraoperative complications, length of surgery, post-operative pain feedback, post-operative length-of-stay (pLOS), and diagnostic yield. Thus, considering BMI ≥ 30 and BMI = 18.5–24.9 as cut-off values, two different groups were created for obese (Group OB) and normal-weight patients (Group NW) for comparison (7). To ensure a more accurate comparison and analysis, we included overweight patients (BMI = 25–29.9) in a separate cluster (Group OW). Baseline characteristics and pre-operative conditions, such as those reported in Table 1, were initially evaluated to ensure acceptable homogeneity between groups. The comorbidities of enrolled patients were scored using the modified Charlson Comorbidity Index (CHC) (8) and the American Society of Anesthesiologists (ASA) score. Pre-operative arterial blood gas analysis was used to measure the patient's partial pressure of oxygen (PaO_2_), while the diffusion lung CO percentage (DLCO%) and forced expiratory volume in the 1st-second percentage (FEV1%) were considered for pulmonary function assessment.

**Table 1:** Baseline pre-operative patients' characteristics.

**Variables**		**Overall (*n* = 202)**	**Group OB (*n* = 40)**	**Group OW** **(*n* = 93)**	**Group NW (*n* = 69)**	***p*-value**
Males, *n* (%)		142 (70.3)	29 (72.5)	66 (71.0)	47 (68.1)	0.87
Median (IQR) age, years		67.4 (60.0–73.5)	70.7 (63.7–75.1)	67.6 (62.3–73.09	64.5 (53.8–73.5)	0.04^a^
Mean (SD) BMI, kg/m^2^		26.8 (4.5)	33.5 (2.5)	27.3 (1.4)	22.2 (2.2)	<0.0001
Median (IQR) CHC index		3 (1–4)	2.5 (1–4)	3 (1–4)	3 (0–4)	0.69
Mean (SD) ASA score		2.4 (0.6)	2.5 (0.6)	2.5 (0.6)	2.3 (0.7)	0.34
Mean (SD) FEV1, %		86.3 (22.7)	86.4 (16.9)	86.5 (23.8)	86.0 (24.5)	0.99
Median (IQR) DLCO, %		56 (47–71)	58.5 (47–73)	56 (46–67)	55.5 (48–72)	0.87
Mean (SD) ABG—PaO_2_, mmHg		76.7 (11.7)	73.3 (8.9)	77.2 (12.6)	78.2 (12.2)	0.20
Smoking habit, *n* (%)	Active smokers	8 (6.8)	0 (0.0)	3 (5.6)	5 (11.9)	0.27
	Ex-smokers	78 (66.1)	16 (72.7)	39 (72.2)	23 (54.8)	
	No smokers	32 (27.1)	6 (27.3)	12 (22.2)	14 (33.3)	

*OB, obese; OW, overweight; NW, normal-weight; BMI, Body Max Index; CHC, Charlson Comorbidity Index; ASA, American Society of Anesthesiologists; FEV1, %, Forced expiratory volume in the 1st-second percentage; DLCO, %, Diffusion lung CO percentage; ABG—PaO_2_, Arterial Blood Gas analysis—Partial pressure of Oxygen.*

a *Group OB vs. Group NW p-value = 0.02*.

According to the modified Clavien–Dindo classification (9), post-operative complications were graded as I–V. Grade I comprised deviation from standard post-operative courses but needed no therapeutic intervention or only brief medication, and grade V represented the patient's death. The length of surgery was calculated as being that comprised between initial skin incision and the completion of its closure. A numeric pain rate scale (NPRS) was used to obtain a score, from zero to 10, representative of post-operative patient discomfort, from readmission to the ward until discharge. Finally, early mortality was defined as mortality within the first 30 days after surgery.

### Surgical Procedure, Post-operative Management, and Follow-Up

As shown in the video of a typical NIAVTS SLB (Supplementary Material), all procedures were performed with the patient placed in the lateral decubitus position and assisted with a nasal cannula or face mask for oxygen supply. A laryngeal mask airway and bronchial blocker were prepared in case of conversion to general anesthesia. The standard anesthesia management was midazolam (0.10–0.15 mg/kg), while conscious sedation with total intravenous anesthesia with propofol and remifentanil was preferentially administered to prevent patient discomfort and to improve patient compliance during the operation. Surgical access was preceded by the skin and subcutaneous local infiltration with lidocaine 2%. In our series, an intraoperative percutaneous intercostal block (performed with thoracoscopic visualization immediately after surgical access), a pre-operative thoracic epidural anesthesia, or only chest wall local anesthesia was applied according to patient consent and preference of the anesthesia team. The surgical technique, varying from uniportal 3-centimeter thoracic incision to multiport VATS approach, depended on the expertise and confidence of each surgeon. Systematically, we performed up to three lung biopsies with wedge resections on the lung regions chosen to be the most suitable for diagnosis, according to the radiological findings pre-operatively discussed by the multidisciplinary team with pulmonologists and radiologists. A chest tube (CT) was placed through the surgical access for pleural drainage and lung re-expansion at the end of the procedure. Patient feedback regarding the procedure was evaluated according to the NPRS report, constantly updated from readmission to the ward until discharge. The patient was declared dischargeable after removing the CT, which was performed without air-leak, checked with the CT under suction, with <200 mL/24 h of serous output, and a permissive chest x-ray imagine.

### Statistical Analysis

Qualitative variables were described with absolute and relative (percentage) frequencies, whereas quantitative variables were summarized with means (standard deviations, SD) or medians (interquartile ranges, IQR) in case of parametric and non-parametric distribution, respectively. Shapiro-Wilk test was used to assess the normal distribution. Qualitative variables were compared using chi-square or Fisher's exact tests (10), whereas ANOVA and Kruskal-Wallis tests were used to compare parametric and non-parametric quantitative variables, respectively. Linear regression analysis was performed to assess the relationship between demographic and clinical variables and quantitative outcomes. Logistic regression analysis was carried out to assess the relationship between BMI groups, intraoperative, and post-operative complications. A two-tailed *p*-value < 0.05 was considered statistically significant. Most relevant results have been reported in this study, according to statistical and data reporting guidelines for the European Journal of Cardio-Thoracic Surgery and the Interactive CardioVascular and Thoracic Surgery journal (11). StataCorp 2021 Stata Statistical Software Release 17 (College Station, TX: StataCorp LLC) was used for the analysis.

## Results

### Patient Demographics

**Figure 1:**
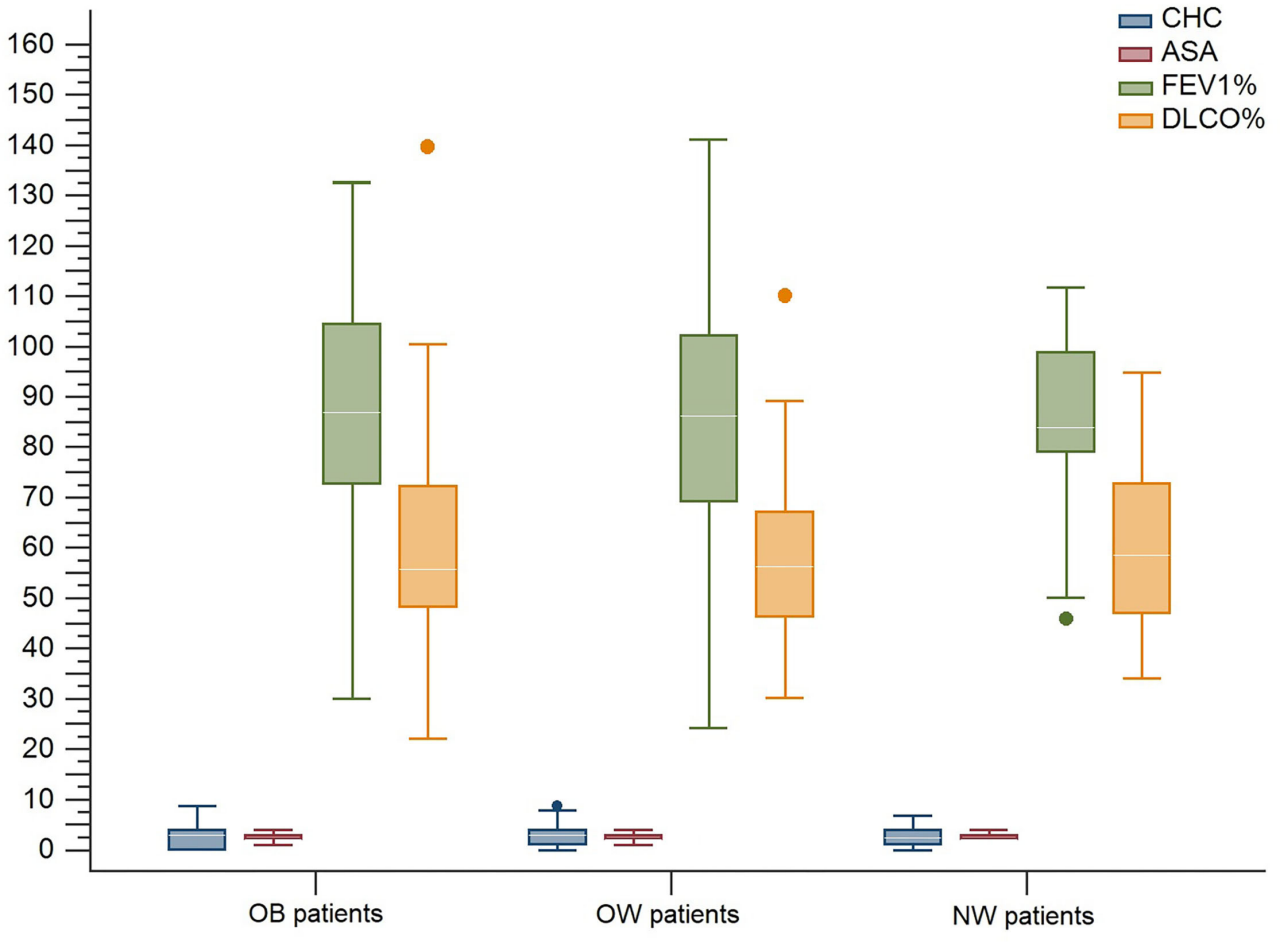
Main pre-operative patient's characteristic distribution. OB, obese; OW, overweight; NW, normal-weight; CHC, Charlson Comorbidity Index; ASA, American Society of Anesthesiologists; FEV1, %, Forced expiratory volume in the 1st-second percentage; DLCO, %, Diffusion lung CO percentage.

Demographics, pre-operative risk factors, and pulmonary function tests were not significantly different between groups (Figure 1).

### Perioperative Findings

As shown in Table 2, the operation time was moderately longer in Group OB (*P* = 0.02). The percentage of intraoperative peripheral capillary oxygen saturation (SpO_2_%) remained satisfactory throughout the operations, with a median (IRQ) of 98 (96, 99). Surgery was completed without conversion to general anesthesia, even in patients with light to moderate pleural adhesions encountered without statistically significant differences (*P* = 0.95). Overall, intraoperative complications occurred in 16 subjects but did not necessitate discontinuing the procedure: persistent cough was the most frequent adverse report, followed by temporary psychomotor agitation, severe pain during surgery, limited respiratory impairment, and minor bleeding.

**Table 2:** Intraoperative clinical characteristics.

**Variables**		**Overall (*n* = 202)**	**Group OB (*n* = 40)**	**Group OW** **(*n* = 93)**	**Group NW (*n* = 69)**	***p*-value**
Side, *n* (%)	Right, *n* (%)	122 (63.2)	25 (64.1)	55 (64.0)	42 (61.8)	0.95
	Left, *n* (%)	71 (36.8)	14 (35.9)	31 (36.1)	26 (38.2)	
Locoregional anesthesia, *n* (%)	Intercostal block, *n* (%)	51 (25.3)	11 (27.5)	26 (28.0)	14 (20.3)	0.64
	TEA, *n* (%)	70 (34.7)	16 (40.0)	29 (31.2)	25 (36.2)	
Median (IQR) length of surgery, min		35 (30–45)	40 (35.0–52.5)	35 (30–45)	35 (25–45)	0.02^a^
Pleural adhesions, *n* (%)		33 (16.3)	6 (15.0)	16 (17.2)	11 (15.9)	0.95
Median (IQR) intraoperative SpO_2_%		98 (96–99)	98 (96–98)	98 (96–99)	98 (97–100)	0.44
Intraoperative complications, *n* (%)		16 (7.9)	4 (10.0)	7 (7.5)	5 (7.3)	0.89
- Minor bleeding		1 (6.3)	0 (0.0)	1 (14.3)	0 (0.0)	
- Cough		10 (62.5)	1 (25.0)	6 (85.7)	3 (60.0)	
- Respiratory impairment		1 (6.3)	1 (25.0)	0 (0.0)	0 (0.0)	
- Psychomotor agitation		3 (18.8)	1 (25.0)	0 (0.0)	2 (40.0)	
- Pain		1 (6.3)	1 (25.0)	0 (0.0)	0 (0.0)	

*OB, obese; OW, overweight; NW, normal-weight; TEA, Thoracic epidural anesthesia; SpO_2_, peripheral O_2_ blood saturation percentage.*

a *Group OB vs. Group NW p-value = 0.01; Group OW vs. Group NW p-value = 0.03*.

### Post-operative Outcomes

Post-operative complication rates were equal, without readmissions or early mortality records (Table 3). Overall, 18 patients had minor post-operative complications (Grade I according to Clavien-Dindo), such as fever and chest X-ray findings of postresectional residual apical pleural space or limited pleural effusion. Two patients needed a surgical revision due to persistent air-leak.

**Table 3:** Post-operative outcomes.

**Variables**		**Overall** **(*n* = 202)**	**Group OB (*n* = 40)**	**Group OW** **(*n* = 93)**	**Group NW (*n* = 69)**	***p*-value**
Median (IQR) post-operative NPRS score		1 (1–2)	2 (1–3)	1 (1–2)	1 (0, 2)	0.45
Biopsy site, *n* (%)						
- RUL		12 (6.2)	2 (5.1)	7 (8.1)	3 (4.4)	0.84
- ML		4 (2.1)	2 (5.1)	2 (2.3)	0 (0.0)	
- RLL		33 (17.1)	7 (18.0)	13 (15.1)	13 (19.1)	
- RUL + RLL		20 (10.4)	6 (15.4)	8 (9.3)	6 (8.8)	
- RUL + ML		3 (1.6)	1 (2.6)	0 (0.0)	2 (2.9)	
- ML + RLL		6 (3.1)	1 (2.6)	3 (3.5)	2 (2.9)	
- LUL		29 (15.0)	5 (12.8)	11 (12.8)	13 (19.1)	
- LLL		20 (10.4)	3 (7.7)	11 (12.8)	6 (8.8)	
- LUL + LLL		22 (11.4)	6 (15.4)	9 (10.5)	7 (10.3)	
- RUL + ML + RLL		44 (22.8)	6 (15.4)	22 (25.6)	16 (23.5)	
Post-operative complications, *n* (%)		22 (10.9)	4 (10.0)	11 (11.8)	7 (10.1)	0.93
- Clavien-Dindo grade I		18 (8.9)	4 (10.0)	8 (8.6)	6 (8.7)	
- Clavien-Dindo grade II		2 (1.0)	0 (0.0)	1 (1.1)	1 (1.4)	
- Clavien-Dindo grade III		2 (1.0)	0 (0.0)	2 (2.1)	0 (0.0)	
Diagnostic yield, *n* (%)	200 (99.0)	39 (97.5)	93 (100)	68 (98.5)	0.38
Diagnostic report, *n* (%)	UIP	101 (50)	24 (60)	50 (53.8)	27 (39.1)	0.03
	Other	99 (49)	15 (37.5)	43 (46.2)	41 (59.4)	
	No diagnosis	2 (1)	1 (2.5)	0 (0.0)	1 (1.5)	
Median (IQR) pLOS, days	2 (1–3)	2 (1–3)	2 (1–3)	2 (1–3)	0.96

*NPRS, Numeric Pain Rate Scale; RUL, right upper lobe; ML, middle lobe; RLL, right lower lobe; LUL, left upper lobe; LLL, left lower lobe; UIP, usual interstitial pneumonia; pLOS, post-operative Length of Stay*.

The procedure was well accepted by all the patients with a median NPRS score of 1 (1, 2). Paracetamol and non-steroidal anti-inflammatory drugs were the only medications administered pro-re-nata. The overall median pLOS was 2 days (1, 3) without a statistically significant difference between groups.

Pathological diagnosis from surgical specimens was successfully obtained in 200/202 (99%) patients: the UIP pattern was observed in 101 exams (50.5%). No statistically significant difference was found considering the distribution of lung biopsy sites between the three groups.

A statistically significant increase in surgery time (beta: 0.71) was observed when locoregional anesthesia was performed (Table 4). Additionally, the increase of a single point of DLCO% resulted in a surgical time reduction (beta: 0.02).

**Table 4:** Contribution of most representative explicative variables in explaining the main outcomes.

**Variables**			
**Intraoperative complications**		**OR (95% CI)**	***p*** **-value**
Groups	Normal-weight	Ref.	Ref.
	Overweight	1.04 (0.32; 3.43)	0.95
	Obese	1.42 (0.36; 5.63)	0.62
**Post**-**operative complications**		**OR (95% CI)**	***p*** **-value**
Groups	Normal-weight	Ref.	Ref.
	Overweight	1.19 (0.44–3.24)	0.74
	Obese	0.98 (0.27; 3.59)	0.98
**Length of surgery, minutes**		**Beta (95% CI)**	***p*** **-value**
Females	0.05 (−0.59; 0.69)	0.87
Age, years	−0.02 (−0.04; 0.006)	0.15
BMI, kg/m^2^	−0.03 (−0.10; 0.03)	0.35
Groups	Normal-weight	Ref.	Ref.
	Overweight	0.06 (−0.60; 0.73)	0.85
	Obese	−0.20 (−0.03; 0.63)	0.64
DLCO, %	−0.02 (−0.04; −0.004)	0.02
Adhesions	0.28 (−0.52; 1.07)	0.50
Locoregional anesthesia	0.71 (0.11–1.30)	0.02
Intraoperative SpO_2_, %	−0.02 (−0.12; 0.09)	0.72
**Post**-**operative length of stay, days**		**Beta (95% CI)**	***p*** **-value**
Females	0.02 (−0.59; 0.63)	0.94
Age, years	−0.014 (−0.04; 0.006)	0.18
BMI, kg/m^2^	−0.04 (−0.10; 0.03)	0.26
Groups	Normal-weight	Ref.	Ref.
	Overweight	0.17 (−0.47; 0.80)	0.61
	Obese	−0.19 (−0.99; 0.60)	0.63
FEV1, %	−0.014 (−0.027; 0.001)	0.03
Locoregional anesthesia	0.68 (0.11–1.24)	0.02
Post-operative complications	3.16 (2.37, 3.95)	<0.0001
**NPRS score**		**Beta (95% CI)**	***p*** **-value**
Groups	Normal-weight	Ref.	Ref.
	Overweight	−0.01 (−0.56; 0.54)	0.97
	Obese	0.29 (−0.38; 0.96)	0.39

*BMI, body mass index; DLCO, %, diffusion lung CO percentage; SpO_2_, peripheral O_2_ blood saturation percentage; FEV1, %, forced expiratory volume in the 1st-second percentage; NPRS, numeric pain rate scale*.

The length of stay was inversely associated with FEV1% (*P* = 0.03), showing a reduction of pLOS (beta: 0.014) every point of increase of FEV1%. Conversely, post-operative complications were associated with an increase of pLOS (beta: 3.16). Similarly, the application of locoregional anesthesia seemed to increase the in-hospital stay (beta: 0.6).

## Discussion

Our study results seem to confirm the safety of NIVATS in ILD patients requiring histological diagnosis with surgical lung biopsy, even when approaching patients with morbid obesity. The VATS technique and analgosedation management guaranteed intraoperative patient comfort and perceived operative safeness, considering the absence of early post-operative mortality and the low incidence of intraoperative or post-operative complications without anesthesia or surgical conversions.

A smaller operating space, the greater excursions of the diaphragm and the major thickness of the chest wall, which could also represent a hindrance, did not seem to represent an issue if considering the comparable length of surgery in obese and in non-obese patients. In addition, the diagnostic yield of NIVATS lung biopsy in obese patients was almost comparable to that in the normal-weight group, and the overall diagnostic rate of this procedure was similar to that reported by traditional surgical techniques under general anesthesia (88.2%; 95% CI, 86.9–89.4%) (12). To date, other Authors described the positive outcomes of NIVATS SLB when compared with mechanically ventilated VATS procedure, mainly for morbidity and pLOS without any significant decrease of the diagnostic yield in patients who underwent tubeless management (13). The negative association between locoregional anesthesia and surgical time could be explained by the extra time taken by the surgeon in performing the percutaneous intercostal block after skin incision. A thorough future evaluation will be warranted to explain this finding and the apparent inverse association of locoregional anesthesia with pLOS.

As part of the correct assessment of the applicability of the NIVATS technique in lung surgery for minor procedures such as lung biopsies in ILD patients, clear indications and selection criteria are lacking. The selection of candidates and the choice of procedures are currently left to local teams based on their institutional case-mix. In major surgical non-intubated thoracic procedures, BMI ≥ 30 is often considered an absolute risk factor as well as difficult intubation or anticipated complex airway management, chronic obstructive pulmonary cases with abundant airway secretions, patients with neurological disorders or unable to cooperate in the awake setting, extensive pleural adhesions or previous pulmonary resections, elderly and fragile patients with severe hypoxia (PaO_2_ <60 mmHg) or hypercapnia (PaCO_2_ > 50/55 mmHg), previous induction chemo- or chemo-radiotherapy, multi-level calcified lymph nodes, and anatomical variations (6, 14, 15).

Previous manuscripts did not agree on obesity as an exclusion criterion for NIVATS lung biopsy. The first case-series of Pompeo et al. excluded obese patients because at high risk (16–19). In other studies, BMI has neither been considered in assessing risk stratification for this set of patients (20–22). Finally, in the preliminary study published by our team, the BMI showed an inverse correlation with the surgical time and the global operating room time for NIVATS SLB procedures, without reporting deeper argumentations (23).

### Limitations and Future Directions

Our study is retrospective and based on relatively small sample size, limiting the inference of the findings. The small sample size did not allow the subdivision of obese patients into appropriate risk classes for a more accurate analysis (7). Nevertheless, since all published studies on this topic were case series or case-control studies, high-quality prospective studies would be needed in the future.

## Conclusion

NIVATS lung biopsy is safe, is associated with low morbidity, and reduces post-operative discomfort and the length of hospital stay, even in obese patients. However, there is a lack of evidence addressing obesity as a negative predictor of outcomes in patients undergoing non-intubated VATS lung biopsy. These results could motivate further interest in gaining more experience to promote such interventions, assess valid and predictable risk factors and produce evidence-based guidelines for the wide spreading of technique and its applications.

## Data Availability Statement

The raw data supporting the conclusions of this article will be made available by the authors, without undue reservation.

## Ethics Statement

The studies involving human participants were reviewed and approved by Ethical Committee of the University of Cagliari. The patients/participants provided their written informed consent to participate in this study. Written informed consent was obtained from the individual(s) for the publication of any potentially identifiable images or data included in this article.

## Author Contributions

RC and PF contributed to the conception and design of the study. FI and LB organized the database. LS and GS performed the statistical analysis. PF wrote the first draft of the manuscript. RC, FG, GG, MP-S, AM, GR, PL, FI, LB, LS, AF, EF, and GS wrote sections of the manuscript. All authors contributed to manuscript revision, read, and approved the submitted version.

## Conflict of Interest

The authors declare that the research was conducted in the absence of any commercial or financial relationships that could be construed as a potential conflict of interest.

## Publisher's Note

All claims expressed in this article are solely those of the authors and do not necessarily represent those of their affiliated organizations, or those of the publisher, the editors and the reviewers. Any product that may be evaluated in this article, or claim that may be made by its manufacturer, is not guaranteed or endorsed by the publisher.
